# Prevalence of Hepatitis B Virus Seromarkers in Young Adults Vaccinated at Birth; Impact on the Epidemiology of Hepatitis B Infection in Iran

**DOI:** 10.5812/hepatmon.17263

**Published:** 2014-05-01

**Authors:** Hiva Saffar, Abolghasem Ajami, Mohammed Jafar Saffar, Jalil Shojaei, Maryam Sotudeh-Anvari, Kiarash Shams-Esfandabad, Ali Reza Khalilian

**Affiliations:** 1 Department of Pathology, Shariaty Hospital, Tehran University of Medical Sciences, Tehran, IR Iran; 2 Department of Immunology, Mazandaran University of Medical Sciences, Sari, IR Iran; 3 Pediatric Infectious Diseases Ward, Boali-Cina Hospital, Mazandaran University of Medical Sciences, Sari, IR Iran; 4 Provincial Center for Diseases Control and Prevention, Mazandaran University of Medical Sciences, Sari, IR Iran; 5 Department of Statistics, Mazandaran University of Medical Sciences, Sari, IR Iran

**Keywords:** Hepatitis B, Iran, Hepatitis B Vaccine, HB Immunogenicity

## Abstract

**Background:**

The epidemiological impact and the duration of protection provided by infant hepatitis B (HB) vaccination are unknown.

**Objectives:**

This study was designed to determine the hepatitis B virus (HBV) infection seromarkers in young adults who have been vaccinated against HBV as the first group of Iranian neonates during 1993 and 1994.

**Patients and Methods:**

We recruited 510 young adults with a history of complete HB vaccination at birth. HBV seromarkers (HB surface antigen (HBs Ag), antibody against HBs Ag (Anti-HBs), and antibody against HB core antigen (Anti-HBc) were measured using ELISA method. Anti-HBs titers ≥ 10 IU/L were considered protective and titers more than 300 IU/L were indicative of a natural boosting. Positive results for Anti-HBc and HBs Ag were considered as breakthrough infection and possible vaccine failure, respectively. The history of acute symptomatic clinical hepatitis was also investigated.

**Results:**

Anti-HBs seropositivity rate was detected in 224 of 510 [95% CI: 39-47] young adults. Breakthrough infection (positive sera for Anti-HBc without chronic infection) was observed in 18 [95% CI: 2.5-3.5] subjects. There were neither HBs Ag positive results nor symptomatic hepatitis cases.

**Conclusions:**

The study results indicated that the neonatal HBV immunization induced a long-term protection against HBV and was very efficacious in reducing chronic HBV infection rate in vaccinated young adults in Iran.

## 1. Background

Globally, it is estimated that at least 2 billion people worldwide have been infected with hepatitis B virus (HBV) and more than 360 million are chronically infected and at risk of serious complications as well as early death. Humans are the only reservoir of HBV and the main routes of HBV transmission varies in different areas of the world according to their HBV endemicity (1, 2). In highly endemic areas, HBV spread most commonly from a mother to her infant at birth and/or intrafamilial person to person transmission in early childhood (1-3). The development of chronic hepatitis B (HB) infection associates inversely with the age of infection acquisition; 80-90% of infants are infected perinatally, about 30-50% of children are infected before the age of 5 and less than 5% occurs in asymptomatic adults (4). A comprehensive approach to eliminating HBV transmission must address perinatally acquired infection and/or infection during early childhood. Universal immunization of newborn infants is the best cost-effective strategy to prevent HB infection and its consequences (5-7). A number of long-term follow-up studies from various epidemiological settings have confirmed that HBsAg carrier status or clinical hepatitis B rarely occurs among successfully vaccinated individuals (8-15).

In Iran, the exact main route of HB infection transmission is unknown. It was supposed that perinatal and early childhood intrafamilial spread might be one of the most common routes of transmission (16, 17). However, during recent years it seems that the horizontal transmission during adulthood has become the main route of transmission in Iran (18). Iran applied the WHO Global Advisory Group Recommendation (19) and neonatal HB vaccination became a part of EPI program in Iran; hence, all newborn infants have been immunized based on a 3-dose schedule. The results of several different studies in Iran revealed that neonatal HB vaccination was highly immunogenic and provided long-term immunity against HB infection in the vaccinees (20-24). This program was expected to affect the HB infection prevalence rate throughout Iran and to decrease the rate of infection after a while. Although, more recent studies and meta-analysis have reported a wide range of HB infection prevalence rates of 1.2% and 9.7% in different parts of Iran (25-28), the result of comparative studies among some groups of adult population and reviews showed a decreasing trend in HBsAg prevalence in general and especially among children and adolescents (29-35). However, the number of studies to evaluate the impact of vaccination on the HBV epidemiology in general (29-31) and/or in the immunized children were rather few (24, 36).

## 2. Objectives

This study was designed and performed to assess the impact of mass neonatal HB vaccination on HB infection prevalence rate in young adults who had been vaccinated 20 years ago in Iran.

## 3. Patients and Methods

We examined the prevalence of HBsAg in vaccinated youth, the number of subjects who retained their anti-HBs, and the incidence of asymptomatic breakthrough infection as well as clinical HB infection occurred in those vaccinated 20 years ago in Iran. The study population consisted of the young adults of west Mazandaran born between March 1993 and December 1994 as the first group of neonates involved in the national HB vaccination program launched in Iran since March 1993. For this study, all subjects were recruited through local public health centers staffs, and the history of their complete primary vaccination was verified in their booklets and medical records. Those patients who were acutely ill, immunocompromised, had chronic renal failure, received no additional HB vaccine, or were recipient of IG, blood, or blood products within preceding three months were excluded. Written informed consent was obtained from all participants and their parents. The Ethics Committee of the Mazandaran and Tehran Universities of Medical Sciences approved the study protocol. An enrolment questionnaire was completed for the record of date of birth and gender, their phone number and address, vaccination record, and history and any evidence of acute clinical or chronic liver disease in the vaccinees as well as their household members. Blood samples were obtained from all the subjects and the collected sera were stored at -20°C for later testing of HBV seromarkers including hepatitis B surface antigen (HBsAg), antibodies to HBsAg (anti-HBs), and antibodies to hepatitis B core antigen (anti-HBc) by ELISA. Serum anti-HBs levels were measured using an ELISA quantitative method (ِDIA.PRO, Diagnostic Bioprobes SrI. Milano-Italy) and their concentrations were expressed as IU/L. sera HBsAg and anti-HBc were detected using qualitative ELISA kits (ِDIA. PRO, Diagnostic Bioprobes SrI. Milano-Italy) according to manufacturer’s instructions.

The individuals with anti-HBs titer ≥ 10 IU/L were defined as vaccine-derived immune, concentrations of 10-99 IU/L as low, and 100-299 IU/L as high levels of protection. The titers of ≥ 300 IU/L were considered the possible result of a recent natural boosting and not discriminated, therefore, the mean anti-HBs concentration was not calculated for them. The subjects who were had positive results for HBsAg were considered HB infected and were further evaluated for possible chronic HB infected (chronic carrier state). The individuals with positive results for both anti-HBc and anti-HBs were defined as having immunity derived from natural HB infection (breakthrough infection) while those with positive results for only anti-HBc were considered possible HB infected and underwent further evaluation for HBV-DNA. All sera with positive results for HBsAg and anti-HBc were rechecked. The collected data were analyzed using descriptive statistical method and 95% of confidence interval (95% CI) was calculated. Chi-square test was used to compare the collected data when was appropriated. A P value < 0.05 was considered statistically significant.

## 4. Results

We included 510 young adults in the study of whom 266 (52%) were females. There was no significant difference between gender (P: NS). The mean age of the subjects was 19.82 years and ranged from 18.8 to 20.5 years. Anti-HBs concentration of more than 10 IU/L was detected in 224 [95% CI: 39-47] subjects. Anti-HBc was found in the sera of 18 [95% CI: 2.5-3.5] of individuals of whom 12 (66.6%) were accompanied by anti-HBs because of an asymptomatic/recovered HB infection (breakthrough infection) and 6 (33.3%) had positive results for anti- HBc alone. To determine either possible silent HB infection or simply a case of asymptomatic breakthrough infection that had lost their positive anti-HBs titer overtime, further investigation is underway. In this study, no young adults had HBsAg positive result, and no participants had reported any evidences of acute symptomatic clinical hepatitis or chronic liver diseases. In addition, in this study, very high levels of anti-HBs (≥ 300 IU/l) unrelated to additional HB vaccination or HB infection were detected in 96 [95% CI: 17-19] subjects. These high titer antibodies were likely caused by natural boosting. The main findings and the relative frequency of seroprotected individuals with different concentrations of anti-HBs are shown in Table 1.

**Table 1.** Hepatitis B Virus Seromarkers and Clinical Hepatitis in 510 Youth ^a^

	Results, No (%)
**Anti-HBS, No. (95%CI)**	
Negative	286 (53, 59)
Positive	224 (39, 47)
Titer 10-99	89 (18, 19)
**Pos**itive** Anti-HBC, No. (95%CI)**	18 (2.5, 3.5)
**Natural boosting, No. (95%CI)**	96 (17, 19)
**HBS Ag**	Not detected
**Clinical Hepatitis**	Not reported

^a^ Youth who were vaccinated with three doses of recombinant hepatitis B vaccine at birth at their 20 years of age in Mazandaran province, IR Iran 2013.

## 5. Discussion

Herein we reported the impact of neonatal HB vaccination on HB infection seromarkers in Iranian youth fully vaccinated at birth. Twenty years after the national HB immunization, nearly 44% of vaccinees retained their seroprotection against HB infection. No participant reported any evidences or signs/symptoms compatible with acute clinical hepatitis, and no one developed a chronic HB infection. In addition, the study revealed that a minority (3.53%) of subjects showed a seroindicator of breakthrough infection and high titers of anti-HBs (≥ 300 IU/L) were detected in 19% of vaccinees probably as a result of natural boosting.

In recent decades, a series of long-term follow-up studies among the vaccinated infants has established that the universal immunization with HB vaccine starting at birth has dramatically reduced the subsequent development of chronic HB infection in young adults from perinatally or early childhood exposure to HBV. These have become apparent in terms of reduction not only in the incidence of acute clinical hepatitis B, but also in HBsAg carrier status among successfully immunized populations (8-15). These beneficial effects were observed in both high-endemic (8-10) and in low-endemic HB infection region (11-15). A long-term follow-up prospective study on vaccinated children from born HBsAg and HBeAg positive mothers and mothers without carrier state in Thailand, a highly endemic country, was performed (8). During the 20-year follow-up, none of the subjects acquired HB infection that evolved chronically and none of the subjects reported clinical symptoms of HB disease. However, during the first decade of study period, possible subclinical breakthrough HB infection accompanied by emergence of anti-HBc was observed in 12.2% of the subjects born to mothers with positive results for both HBsAg and HBe Ag. Furthermore, during the second decade, possible subclinical breakthrough infection was detected in 12.8% of all vaccinated groups. Increase in anti-HBs concentrations unrelated to additional HB vaccination or HB infection was detected in 10% of subjects in the first decade and 10.7% in the second decade of life. These increases in anti-HBs levels not accompanied by anti-HBc were likely caused by natural boosting. Similarly, during a longitudinal study in fully vaccinated infants in China (10), yearly blood samples were collected. During the 22-year study period, no case of Hepatitis evolving chronically was detected and 23% of subjects had anti-HBs responses without any additional booster vaccination. Although most of these studies were conducted in region with high endemicity, the data from countries with low endemicity also showed beneficial impact on HBV prevalence (10-15). For example, a study from Catalonia, a low-endemic area in Spain (13), revealed that fifteen years after the introduction of preadolescent HB vaccination and vaccination of the infants of HBsAg positive mother, the HBsAg carrier state decreased from 1.5% to 0.7% and anti-HBc prevalence rate declined from 15.6% to 8.7%. Similar to this pattern was reported from Italy, a country with low to intermediate endemicity, where a universal HB vaccination was launched in 1991 for infants as well as for the adolescents. Surveillance data showed an overall decrease in the incidence of acute clinical hepatitis and chronic carrier state (14, 15).

After the introduction of universal vaccination program in Iran, many different studies and reviews were done to determine HB infection prevalence rates and HBsAg carrier status as well as its changing trend. Most of these studies were performed on the adult population and/or among of special groups (17, 29-35). However, the number of studies to evaluate the impact of vaccination on HB infection epidemiology among general populations and/or vaccinated individuals were rather few (24, 29-31, 36). The first nationwide comparative assessment of program on HBsAg prevalence rate was carried out five years after the implementation of the universal HB vaccination in Iran (29). In this large nationwide populations-based survey on a representative sample of 1/1000 of the country population, HBsAg carrier state rate among the different age groups of older than two years were investigated. The results indicated that the overall HBsAg seropositivity rates showed no significant changes between the years 1991 (17) and 1999 (29): 1.7% vs 1.7% (two years before and five years after the introduction of the programs respectively). However, the HBsAg prevalence rate decreased significantly from 1.3% to 0.8% (P < 0.05) in the age group 2-14 years including some vaccinated children. Furthermore, to evaluate the HBsAg prevalence rate in general population, two similar cross-sectional studies were carried out ten years apart in Khorasan province (30) and Mashhad city (31). In the first study (30) (six years after implementing universal program in Iran), 4528 individuals older than two years of age were included. Study results indicated that the overall HBsAg prevalence rate was 3.6%; however, this rate among two- to 20-year-old subjects including some vaccinated children was 2.45%. In the second study (31) (16 years after beginning of the program), 1678 individuals older than one year of age from Mashhad city (center of Khorasan province) were studied for HBsAg status. In comparison with the earlier study, the overall HBsAg prevalence rate was decreased markedly from 3.6% to 1.39%. However, the highest rate of prevalence (2.98%) was observed in those older than 35 years of age, and the lowest rate (0.24%) was reported in those younger than 15 years of age. All of these studies were carried-out on general population including some vaccinated children; hence, it did not evaluate the effect of program on HBV epidemiology. However, to assess the long-term impact of vaccination on HB infection rates in fully vaccinated children, the first study was performed on 146 fully vaccinated ten-year-old children (36). The study revealed that 47.9% of immunized children retained their vaccine-induced seroprotection, 7.5% had positive anti-HBc results indicating breakthrough infection, and none of the vaccinees became HBsAg carrier. In addition, in another study on 453 ten-year-old fully vaccinated children, long-term immunity, immunological memory, and possible chronic HB infection were studied (24). Overall, 57.8% of the vaccinated children had anti-HBs positive results. Those with anti-HBs levels less than 10 IU/L with one dose of HB vaccine were vaccinated. All boosted children showed an anamnestic response indicating immunological memory and no one was diagnosed as chronically HB infected (24).

The present study findings along with the data collected by earlier studies from Iran (24, 29-31, 36) are in accordance with those reported worldwide (8-15) and provided additional evidence and confirmed long-term efficacy of Iranian HB immunization programs in decreasing HB disease and carrier status rates in the vaccinated individuals and probably the general population for more than twenty years. In this regard, a meta-analysis by Poorolajal et al. was performed on long-term follow-up studies including 9356 vaccinees. Results indicated that overall cumulative incidence of HB breakthrough infection was 0.007 (95% CI: 0.005-0.010) five to 20 years after primary vaccination. Moreover, the protection provided by a full course of vaccine beginning at early infancy lasted for at least two decades. To determine vaccine efficacy for longer periods and possible need of booster dose, additional studies were recommended (37, 38). Fifteen to 20 years after primary infant HB immunization, some vaccinees engaged in risky behaviors and/or occupation that might put them at risk of more exposure to HB infection. In this regard, the main question that remains to be answered is "how long vaccine-induced immunity is expected to last?" To answer this question, additional long-term follow-up and surveillance in HB vaccinees in different countries are still needed to establish whether a primary series of vaccination of immunocompetent children confers long life protection without a need for additional booster dose. Study design was cross-sectional and this is the main limitation of study.

The study results revealed that neonatal HB vaccination was highly efficacious in protecting vaccinees against acute clinical hepatitis and chronic HB infection for more than 20 years, even when more than half of the vaccinees lost their seroprotection. However, the durations of protection offered by primary vaccination remained unclear. The need for booster dose in adulthood is still to be determined and continuous follow-up is recommended.
